# Radiomics and Machine Learning Differentiate Soft-Tissue Lipoma and Liposarcoma Better than Musculoskeletal Radiologists

**DOI:** 10.1155/2020/7163453

**Published:** 2020-01-07

**Authors:** Ieva Malinauskaite, Jeremy Hofmeister, Simon Burgermeister, Angeliki Neroladaki, Marion Hamard, Xavier Montet, Sana Boudabbous

**Affiliations:** Geneva University Hospital, Diagnosis Department, Radiology Division, Rue Gabrielle-Perret-Gentil 4, 1211 Geneva 4, Switzerland

## Abstract

Distinguishing lipoma from liposarcoma is challenging on conventional MRI examination. In case of uncertain diagnosis following MRI, further invasive procedure (percutaneous biopsy or surgery) is often required to allow for diagnosis based on histopathological examination. Radiomics and machine learning allow for several types of pathologies encountered on radiological images to be automatically and reliably distinguished. The aim of the study was to assess the contribution of radiomics and machine learning in the differentiation between soft-tissue lipoma and liposarcoma on preoperative MRI and to assess the diagnostic accuracy of a machine-learning model compared to musculoskeletal radiologists. 86 radiomics features were retrospectively extracted from volume-of-interest on T1-weighted spin-echo 1.5 and 3.0 Tesla MRI of 38 soft-tissue tumors (24 lipomas and 14 liposarcomas, based on histopathological diagnosis). These radiomics features were then used to train a machine-learning classifier to distinguish lipoma and liposarcoma. The generalization performance of the machine-learning model was assessed using Monte-Carlo cross-validation and receiver operating characteristic curve analysis (ROC-AUC). Finally, the performance of the machine-learning model was compared to the accuracy of three specialized musculoskeletal radiologists using the McNemar test. Machine-learning classifier accurately distinguished lipoma and liposarcoma, with a ROC-AUC of 0.926. Notably, it performed better than the three specialized musculoskeletal radiologists reviewing the same patients, who achieved ROC-AUC of 0.685, 0.805, and 0.785. Despite being developed on few cases, the trained machine-learning classifier accurately distinguishes lipoma and liposarcoma on preoperative MRI, with better performance than specialized musculoskeletal radiologists.

## 1. Introduction

Lipoma and liposarcoma are soft-tissue tumors of mesenchymal origin, often containing visible fat on MRI examination [1]. Differentiating soft-tissue lipoma from liposarcoma on imaging is crucial for patient management, as their follow-up, treatment, and prognosis drastically differ (ranging from almost 100% 5-year survival for lipoma to 60–70% [2] for liposarcoma). While some radiological features might help identifying liposarcoma (such as size >10 cm, thick septations, globular and/or nodular nonadipose regions, or lesion containing less than 75% fat [3]), a significant number of benign lipoma also have imaging appearance mimicking liposarcoma. The reverse is also true; well-differentiated liposarcoma (WDL), accounting for 50% of common liposarcoma [4], may also resemble ordinary lipomas with similar imaging, making the distinction difficult on MRI. Previous study showed that specialized musculoskeletal (MSK) radiologists could differentiate between lipoma and liposarcoma with only 69% accuracy on MRI [5]. In case of uncertain diagnosis following MRI, further invasive procedure (percutaneous biopsy or surgery) is often required to allow for diagnosis based on histopathological examination.

Radiomics is a method designed to extract a large number of noninvasive, quantitative, and reproducible characteristics from radiological images, thereby enabling data analysis and prediction [6, 7]. Coupled with machine learning (ML) methods, this technique allows for several types of pathologies encountered on radiological images to be automatically and reliably distinguished, potentially increasing diagnostic accuracy and allowing for better outcome for patients [8]. Previous studies using radiomics in soft-tissue lesions showed that it allows the distinction between intermediate and high-grade sarcoma [9] and could predict histopathological grading of soft-tissue sarcoma on preoperative MRI [10]. However, these studies did not investigate whether radiomics could help distinguish benign soft-tissue lipoma from liposarcoma nor the accuracy of radiomics compared to specialized MSK radiologists in such tasks.

The purpose of our study was to train and to assess the ability of a predictive model based on radiomic features coupled with ML methods to distinguish lipoma and liposarcoma on preoperative MRI. We also aimed to compare the prediction accuracy of such radiomics model with those of specialized MSK radiologists.

## 2. Materials and Methods

**Figure 1:**
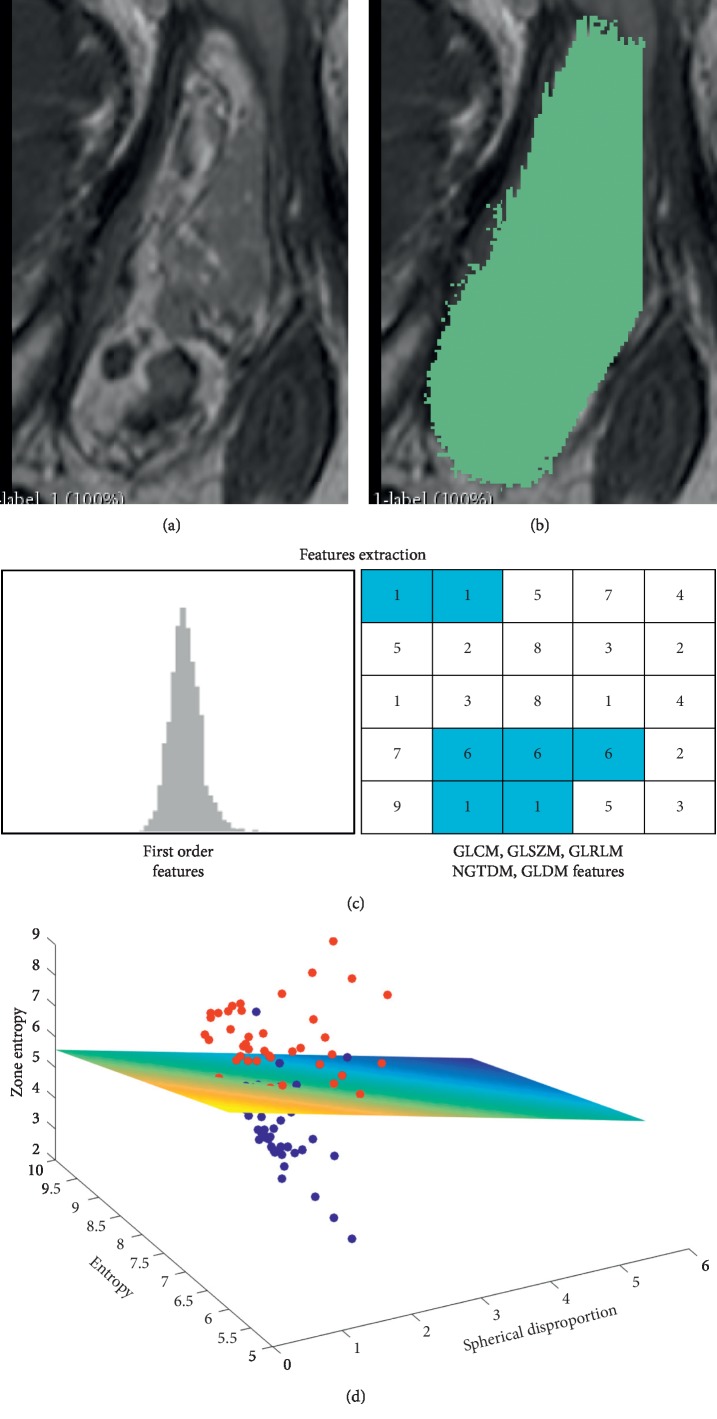
Radiomics analysis pipeline.Radiomics analysis pipeline for all included patients, showing (a) acquisition of the T1-SE image, followed by (b) soft-tissue lesion segmentation using Slicer 3D and (c) radiomics features extraction using Pyradiomics. (d) Radiomics features were finally used to train and assess the performance of a machine-learning classifier to distinguish liposarcoma and lipoma.

The study protocol was approved by the ethical committee of our State, with a waiver of the requirement to obtain informed consent. The requirement for informed consent was waived because (1) the study is retrospective and (2) MRI sequences used here were part of the routine MRI protocol, and the current study did not involve either changes in patient clinical management nor additional diagnostic procedure.  Figure 1 summarizes the different steps of our study.

We remind that atypical lipomatous tumor and well-differentiated liposarcoma are synonyms that are identical morphologically and karyotypically according to the WHO classification (2013) of tumors of soft tissue and bone [4] and the choice of the terminology is determined by the reciprocal comprehension between surgeon and pathologist to prevent impropriate treatment.

### 2.1. Subjects

We retrospectively retrieved from our institutional database all consecutive patients referred to our institution for soft tissue multidisciplinary board between January 2015 and December 2017, to identify those who underwent MRI examination and were further diagnosed with either lipoma or liposarcoma by histopathology (used as the gold-standard for further classification process). Inclusion criteria were (1) patients with soft-tissue lesion referred for investigation for specialized musculoskeletal radiologist of our institution, with imaging protocol including an MRI study with an axial T1-weighted Spin-Echo (T1-SE) sequence less than 1 month before percutaneous biopsy or surgery, (2) patients with diagnosis of soft-tissue lipoma or liposarcoma confirmed by histopathological examination, and (3) patients with no history of surgery or other treatment in the affected area. Exclusion criteria were (1) poor MRI image quality or (2) soft-tissue tumor in retroperitoneal space (as investigated with different protocol at our institution). Thus, we enrolled 38 patients referred for radiological investigation of a soft-tissue lesion further diagnosed as lipoma (*N* = 24) or liposarcoma (*N* = 14, with 6 myxoid liposarcomas, 2 dedifferentiated liposarcomas, 1 atypical spindle cell lipoma and 5 well-differentiated liposarcomas) on subsequent histopathological examination.

### 2.2. Clinical Characteristics of the Patients

The demographics and radiological characteristics of the lipoma and liposarcoma groups are summarized in Table 1. Tumor localization is summarized in Table 2.

### 2.3. MRI Examination and Lesion Segmentation

All included patients underwent MRI on a 3.0 Tesla MRI scanner 3 Tesla Achieva MRI (Philips Healthcare, Netherlands), 3 Tesla Skyra (Magnetom Siemens Healthineers, Germany) with a protocol including axial T1-weighted SE images without contrast enhancement. This T1-weighted SE image was acquired with a slice thickness varying between 2 and 5 mm according to covering area and a resolution between 0.3 and 0.5 mm (spacing between slices from 2.2 to 5.5 mm). MR scanning parameters of the T1-weighted SE image are as follows: thickness 2–5 millimeters (mm), repetition time (TR): 470–832 milliseconds (ms), and echo time (TE): 7–27 ms. The whole MRI session also included T2-weighted fluid-sensitive, diffusion-weighted, and postcontrast fat-saturated T1-weighted sequences, as part of the routine clinical protocol of our institution.

Soft-tissue lesion segmentation was performed by a senior board certified MSK radiologist *∗∗* (10 years of experience in MSK after board-specialization) on T1-weighted SE images, using Slicer 3D (version 4.8.1) [11]. All segmentations were performed using Fast-Grow-Cut algorithm implemented in Slicer 3D, with a manual correction in case of segmentation errors. When segmenting the images, the senior radiologist was blinded to the clinical and pathological diagnosis. Finally, a second radiologist (2 years of experience) performed the same segmentation steps for a subset of the patients (*N* = 12, including 6 malignant lesions), to ensure that our classification results are robust to the segmentation procedure. The classification performance for this subset of patients was compared between both segmentations using intraclass correlation coefficient.

### 2.4. Radiomics Features Extraction

Eighty-six radiomics features were extracted from all segmented soft-tissue lesions (see above) using PyRadiomics (version 1.3.0) [12]. Extracted radiomics features included first-order features, shape features, gray level co-occurrence matrix (GLCM) features, gray level size zone matrix (GLSZM) features, gray level run length matrix (GLRLM) features, neighboring gray tone difference matrix (NGTDM) features, and gray level dependence matrix (GLDM) features (Supplementary [Supplementary-material supplementary-material-1] for details of the extracted features).

### 2.5. Model Construction

Our classification machine-learning model was based on a support vector machine (SVM) classifier and trained on all previously extracted 86 radiomics features. Our model comprised a first standardization step designed to normally distribute radiomic features (with 0 mean and unit variance), followed by a principal component analysis (PCA) to reduce the risk of overfitting and potential redundancy of radiomic features, along with a final classification step using SVM classification algorithms (kernel = linear, penalty parameter *C* = 0.1). The SVM classifier was trained with the first principal components (PCs), explaining 97.0% of the variance, to dampen the risk of overfitting. The classification process was repeated with 2, 10, and all PCs, explaining 99.8%, 99.9%, and 100% of the variance, respectively, which did not modify the results. As our data set contains more benign than malignant lesions, we also repeated the classification process with different class weights to account for class imbalance ((1.71 : 1) as observed ratio of lipoma and liposacroma, as well as [2 : 1] and [1 : 2]), which did not modify the classification performance. Finally, we assessed the performance of three other machine-learning classifiers in distinguishing lipoma from liposacroma, by replacing our SVM with naive Bayes, linear discriminant analysis, or logistic regression algorithms, all with default values and using the same evaluation procedure (Section 2.6).

### 2.6. Model Evaluation

To assess the generalization performance of our predictive machine-learning model, we computed the receiver operating curve (ROC) analysis with Monte-Carlo cross-validation (MCCV) approach, similar to a suggestion by Shi et al. [13]. Monte-Carlo cross-validation randomly selects some fraction of the data to form the training and the testing data set. The process is repeated multiple times (*N* = 325) to generate new training and testing sets: for each iteration, one lipoma and one liposarcoma were randomly selected for validation, whereas the remaining 36 patients were used for training the model. This ensures that standardization, dimension reduction, and machine-learning model training are fit on the training data and then assessed to the testing data, in order to prevent any data leakage. Using the same MCCV method, we were able to compute the area under the ROC curve (AUC), overall accuracy, sensitivity, specificity, and log-loss. Finally, a permutation testing was performed using the same MCCV methods, by randomly shuffling *N*-times (*N* = 10'000) the test set label and by computing the prediction accuracy. This permutation test aimed to obtain the correct prediction distribution under a null hypothesis, while controlling for Type I error. All classification and evaluation steps of the machine-learning model were carried out using the SciKit-learn package (Version 0.19.1) [14].

### 2.7. Comparison between Radiomics and Radiologists' Classification

In order to compare the performance of our radiomics approach with those of specialized radiologist in differentiating lipoma and liposarcoma on MRI, we asked three MSK radiologists 2 (*∗∗*), 5 (*∗∗*), and 10 (*∗∗*) years of specialized MSK experience after board-certification, for radiologists 1, 2, and 3, respectively to classify cases as lipoma or liposarcoma based on the whole MRI session for all patients (see available MRI sequences in Section 2.2). The classification was based on (1) homogeneity/heterogeneity, (2) presence of thick septa, (3) restriction of diffusion if present, and (4) nodular enhancement. We then used a two-tailed McNemar test to compare radiomics model versus the three radiologists independently and versus the consensus made by the three radiologists (i.e., majority voting of the three radiologists). We also computed the classification agreement between radiologists using Kappa statistics.

## 3. Results

### 3.1. Radiomics Model Evaluation

**Figure 2:**
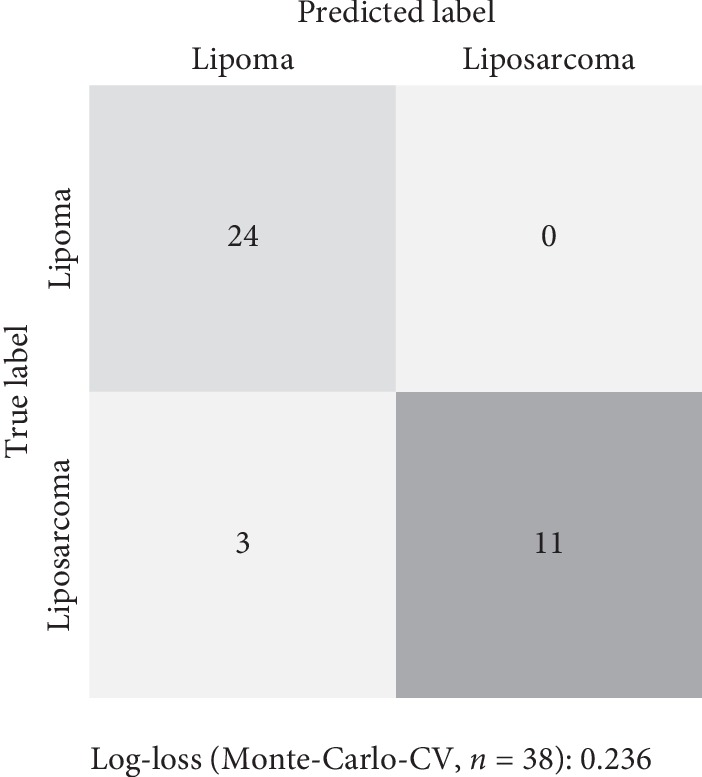
A confusion matrix showing the diagnostic performance of the model.

The radiomics model demonstrated a high level of diagnostic accuracy, at 94.7%. The sensitivity and specificity were 88.8% and 100%, for positive and negative predicting values of 100% and 78.5%. The radiomics model achieved an AUC-ROC of 0.926 (Figure 2 and Table 3). Permutation testing revealed that our machine-learning model performs significantly better than chance (*p* < 0.001), proving that the risk of fortuitous correlation between features and outcomes remains very low. The three other machine-learning algorithms further assessed also yielded classification performances significantly better than chance (*p* < 0.001), with naive Bayes classifier showing a diagnostic accuracy at 79.0% and AUC-ROC:0.809, linear discriminant analysis with an accuracy of 89.5% and AUC-ROC of 0.929, and logistic regression classifier having a diagnostic accuracy of 73.7% and AUC-ROC of 0.812. Finally, we observed good intraclass correlation for the classification of a subset of lesion for which the automatic segmentation was repeated by a second radiologist (ICC = 0.70).

### 3.2. Radiomics and Radiologist's Comparison

Finally, we compared the accuracy of individual radiologists and of the consensus of the three radiologists together to the accuracy of the radiomics model in identifying lipoma and liposarcoma (Tables 4 and 5). We found that the radiomics model performed significantly better than radiologists 1 and 2 (*p* < 0.05) and with a trend to perform better than radiologist 3 and than the group consensus between the three MSK radiologists (*p* < 0.10). Classification agreement between the three radiologists was 0.551 using kappa statistics.

**Figure 3:**
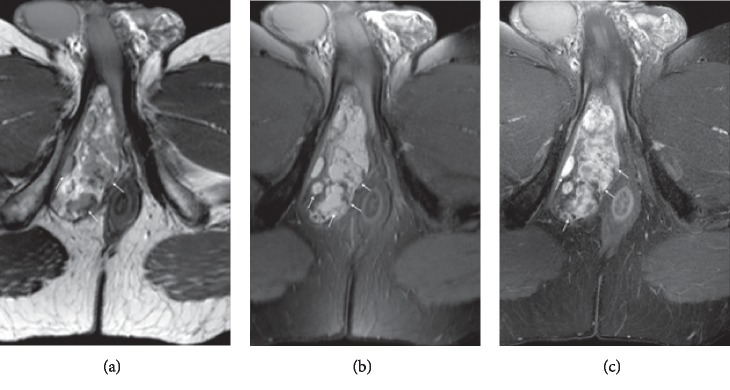
Case of an atypical spindle cell lipoma. Perineal mass in 42-year-old man, diagnosed as suspected of liposarcoma by three radiologists and classified as lipoma by radiomics. Histological analysis concluded to an atypical spindle cell lipomatous tumor, thus corresponding to a low-grade liposarcoma.

Figure 3 is an illustrative example of an atypical spindle cell lipoma, classified as liposarcoma by the three MSK radiologists and as a lipoma by the ML algorithm.

**Figure 4:**
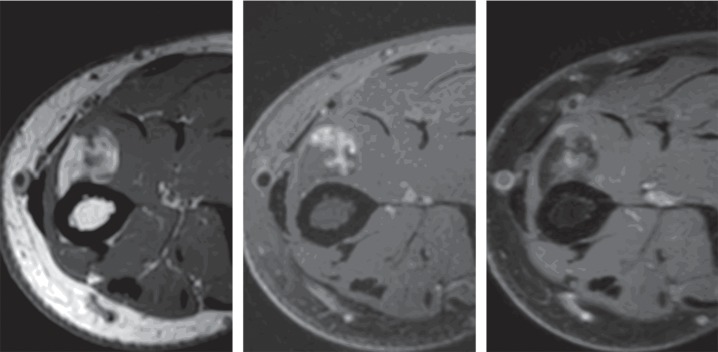
Forearm mass in a 27-year-old man diagnosed as suspected of liposarcoma by three radiologists and classified as lipoma by radiomics. Histological analysis concluded to a lipoma.

Figure 4 is an illustrative example of a lipoma classified as a liposarcoma by the three MSK radiologists and as a lipoma by the ML algorithm. In that case, the ML algorithm outperformed the MSK radiologist.

## 4. Discussion

Distinguishing atypical lipoma from liposarcoma is challenging on conventional MRI examination, and previous study showed that specialized MSK radiologists achieved only 69% accuracy in such task [5]. Here, we show that radiomics coupled with machine-learning methods performed better than specialized MSK radiologist at distinguishing lipoma and liposarcoma on preoperative unenhanced T1-weighted MRI, achieving 94.7% diagnostic accuracy.

Based on morphological sequences, the diagnosis is not always easy as nodular foci, hyperintensity on T2 fat-saturated sequence, thick septa, and nodules on T1 lack of specificity [15]. The presence of mesenchymal components or fat necrosis focus can cause nodular appearance. This overlap [16] explains that despite the usefulness of MRI findings for the preoperative diagnosis, immuno-histochemical tests such as MDM2 and CDK4 should be considered in the majority of cases [17]. The presence (or absence) of MDM2 and CDK4 is gathered by biopsy or resection which may be technically difficult to perform mainly for deep lesions. In addition, the biopsies target in general one site in the tumor, which may be the adipose component, generating sampling errors [18].

In an effort to be reproducible, some teams proposed a score to differentiate lipoma from liposarcoma based on size, depth, septal architecture, and contrast enhancement with an average score of 1.7 for lipoma compared to 5.1 for WDL achieving 100% sensitivity and 77% specificity [2].

Differentiation of lipoma from liposarcoma using texture and shape analysis was performed by Thornhill et al. [19]. They used multiple sequences (T1-weighted, T2-weighted, T2-weighted fat suppressed, short time inversion recovery (STIR), and contrast-enhanced sequences) at 1.5 Tesla. Textural and morphological features extracted from T1-weighted sequences achieved an accuracy of 85%, sensitivity of 96% and specificity of 91% compared to radiologists.

The use of T1-weighted SE sequence in this study relied on the robustness of this sequence. Previous publications have demonstrated that T1-weighted sequences enable stable extraction of features and that texture analysis based on T1-weighted sequences acquired in different machines are similar for the same type of tumor [20, 21]. Juntu showed that machine-learning classifiers trained with texture analysis features extracted from the tumor areas in T1-weighted MR images are potentially valuable tools for the differentiation between malignant and benign tumors and that SVM (support vector machine) performed as good as or better than the radiologists [22].

This study has some limitations. First of all, the number of included patients is limited. Despite the relatively low number of included patients, a Monte-Carlo cross-validation (MCCV) proved that machine learning trained on radiomics features achieved better performances than MSK radiologists. Monte-Carlo cross-validation gives a more accurate estimation of prediction ability [23]. Secondly, the diagnostic performance of radiomics was not compared with other common advanced MRI sequences such as diffusion-weighted imaging, apparent diffusion coefficient, or dynamic contrast enhancement. Future studies using multiparametric MRI should investigate the added values of such sequences [9]. Lastly, no separate cohorts were used to validate the model.

In conclusion, we showed that radiomics and machine-learning allow for good differentiation between lipoma and liposarcoma on preoperative MRI, with better performance than specialized MSK radiologists, potentially decreasing the diagnostic uncertainty in these clinical situations [8]. Further research is however required to determine how radiomics might help reduce the number of invasive procedures for patients with benign soft-tissue lipoma and fasten treatment of those with liposarcoma, allowing for better outcome for both groups of patients.

## Figures and Tables

**Table 1:** Demographic and radiological characteristics of the lipoma and liposarcoma groups.

	Lipoma	Liposarcoma	*p* values
Mean age (±SD)	53.64 (±12.07)	61.6 (±16.63)	0.0715
Male/female	16/8	13/1	0.559
Mean size (±SD)	8.27 (±5.09)	14.02 (±7.23)	0.0032
Location superficial/deep	6/18	2/12	0.092

**Table 2:** Tumor location.

	Lipoma	Liposarcoma
Thigh	10	5
Abdominal wall	2	2
Dorsal wall	2	3
Arm/forearm	4	—
Leg/ankle	3	—
Neck	2	—
Pelvis	1	4

**Table 3:** Accuracies of radiologists and radiomics model in identifying liposarcoma versus lipoma.

	RMX (%)	RAD 1 (%)	RAD 2 (%)	RAD 3 (%)	Consensus (%)
Accuracy	94.7	65.8	81.6	79.0	81.6
Sensitivity	88.8	76.9	76.9	76.9	76.9
Specificity	100	60.0	84.0	80.0	84.0
PPV	100	50.0	71.4	66.7	71.4
NPV	78.5	83.3	87.5	87.0	87.5
AUC	0.926	0.685	0.805	0.785	0.805

RMX: radiomics model; RAD 1, 2, and 3: MSK radiologists 1, 2, and 3; consensus: group consensus between the three MSK radiologists; PPV: positive predicting value; NPV: negative predicting value; AUC: area under the receiver operating curve.

**Table 4:** Diagnosis made by the MSK radiologist and by the ML as compared to pathology.

Pathological diagnosis	Radiologist 1	Radiologist 2	Radiologist 3	ML prediction	ML probability
Lipoma	Lipoma	Lipoma	Lipoma	Lipoma	0.12
Lipoma	Lipoma	Liposarcoma	Lipoma	Lipoma	0.13
Lipoma	Liposarcoma	Lipoma	Lipoma	Lipoma	0.14
Lipoma	Liposarcoma	Lipoma	Liposarcoma	Lipoma	0.12
Lipoma	Lipoma	Lipoma	Lipoma	Lipoma	0.14
Lipoma	Lipoma	Lipoma	Lipoma	Lipoma	0.12
Lipoma	Liposarcoma	Lipoma	Lipoma	Lipoma	0.13
Lipoma	Liposarcoma	Lipoma	Lipoma	Lipoma	0.11
Lipoma	Liposarcoma	Lipoma	Lipoma	Lipoma	0.11
Lipoma	Lipoma	Lipoma	Lipoma	Lipoma	0.13
Lipoma	Lipoma	Lipoma	Lipoma	Lipoma	0.36
Lipoma	Lipoma	Lipoma	Lipoma	Lipoma	0.14
Atypical lipoma	Liposarcoma	Liposarcoma	Liposarcoma	Lipoma	0.13
Lipoma	Liposarcoma	Lipoma	Lipoma	Lipoma	0.14
Lipoma	Lipoma	Lipoma	Lipoma	Lipoma	0.12
Lipoma	Liposarcoma	Lipoma	Liposarcoma	Lipoma	0.08
Lipoma	Lipoma	Lipoma	Lipoma	Lipoma	0.13
Lipoma	Lipoma	Lipoma	Lipoma	Lipoma	0.12
Lipoma	Liposarcoma	Lipoma	Lipoma	Lipoma	0.18
Lipoma	Lipoma	Lipoma	Lipoma	Lipoma	0.14
Lipoma	Lipoma	Lipoma	Liposarcoma	Lipoma	0.13
Lipoma	Lipoma	Lipoma	Lipoma	Lipoma	0.14
Lipoma	Lipoma	Liposarcoma	Lipoma	Lipoma	0.12
Lipoma	Lipoma	Lipoma	Lipoma	Lipoma	0.14
Atypical spindle cell lipoma	Liposarcoma	Liposarcoma	Liposarcoma	Lipoma	0.15
Dedifferentiated liposarcoma	Liposarcoma	Liposarcoma	Liposarcoma	Liposarcoma	0.65
Dedifferentiated liposarcoma	Liposarcoma	Liposarcoma	Liposarcoma	Liposarcoma	0.91
Myxoid liposarcoma	Liposarcoma	Liposarcoma	Liposarcoma	Liposarcoma	0.88
Myxoid liposarcoma	Liposarcoma	Liposarcoma	Liposarcoma	Liposarcoma	0.65
Myxoid liposarcoma	Liposarcoma	Liposarcoma	Liposarcoma	Liposarcoma	0.88
Myxoid liposarcoma	Liposarcoma	Liposarcoma	Liposarcoma	Liposarcoma	0.90
Myxoid liposarcoma	Lipoma	Lipoma	Lipoma	Liposarcoma	0.79
Myxoid liposarcoma	Liposarcoma	Liposarcoma	Liposarcoma	Liposarcoma	0.87
Well differentiated liposarcoma	Lipoma	Lipoma	Lipoma	Lipoma	0.44
Well differentiated liposarcoma	Liposarcoma	Liposarcoma	Liposarcoma	Liposarcoma	0.81
Well differentiated liposarcoma	Liposarcoma	Liposarcoma	Liposarcoma	Liposarcoma	0.78
Well differentiated liposarcoma	Liposarcoma	Liposarcoma	Liposarcoma	Liposarcoma	0.78
Well differentiated liposarcoma	Lipoma	Lipoma	Lipoma	Lipoma	0.11

ML: machine learning.

**Table 5:** Statistical comparison of accuracies of radiologists versus radiomics model using the McNemar test.

	RMX VS RAD1	RMX VS RAD 2	RMX VS RAD 3	RMX VS CONSENSUS
CHI2	9.09	3.20	4.17	3.20
P-VAL	0.003	0.074	0.041	0.074

RMX: radiomics model; RAD 1, 2, and 3: MSK radiologists 1, 2, and 3; consensus: group consensus between the three MSK radiologists.

## Data Availability

The private patient data used to support the findings of this study are restricted by the Swiss Ethics Committees on research involving humans in order to protect patient privacy. Data are available from PD-Dr Sana Boudabbous and Dr Jeremy Hofmeister, University Geneva Hospital, mail: sana.boudabbous@hcuge.ch and jeremy.hofmeister@hcuge.ch, for researchers who meet the criteria for access to confidential data.

## Abbreviations

ADC: Apparent diffusion coefficient
AI: Artificial intelligence
AUC: Area under the curve
CAD: Computed assisted diagnosis
CI: Confidence interval
DCE: Dynamic contrast-enhanced
DWI: Diffusion-weighted imaging
GE: Gradient echo
MRI: Magnetic resonance imaging
RLNU: Run length nonuniformity
ROC: Receiver operating characteristic
ROI: Region of interest
mm: Millimeter
ms: Millisecond
MSK: Musculoskeletal
STIR: Short time inversion recovery
SVM: Support vector machine
TE: Echo time
TR: Repetition time
WDL: Well-differentiated liposarcoma.

## Supplementary Materials

Supplementary Materials Table S1: extracted radiomics features.

## References

[1] Munk P. L., Lee M. J., Janzen D. L. (1997). Lipoma and liposarcoma: evaluation using CT and MR imaging. *American Journal of Roentgenology*.

[2] Nagano S., Yokouchi M., Setoguchi T. (2015). Differentiation of lipoma and atypical lipomatous tumor by a scoring system: implication of increased vascularity on pathogenesis of liposarcoma. *BMC Musculoskeletal Disorders*.

[3] Kransdorf M. J., Bancroft L. W., Peterson J. J., Murphey M. D., Foster W. C., Temple H. T. (2002). Imaging of fatty tumors: distinction of lipoma and well-differentiated liposarcoma. *Radiology*.

[4] Fletcher C. D. M., Bridge J. A., Hogendoorn P. W. C. (2013). *World Health Organization Classification of Tumours of the Soft Tissues and Bone*.

[5] O’Donnell P. W., Griffin A. M., Eward W. C. (2013). Can experienced observers differentiate between lipoma and well-differentiated liposarcoma using only MRI?. *Sarcoma*.

[6] Shaikh F., Franc B., Allen E. (2018). Translational radiomics: defining the strategy pipeline and considerations for application-part 1: from methodology to clinical implementation. *Journal of the American College of Radiology*.

[7] Gillies R. J., Kinahan P. E., Hricak H. (2016). Radiomics: images are more than pictures, they are data. *Radiology*.

[8] Thrall J. H., Li X., Li Q. (2018). Artificial intelligence and machine learning in radiology: opportunities, challenges, pitfalls, and criteria for success. *Journal of the American College of Radiology*.

[9] Corino V. D. A., Montin E., Messina A. (2018). Radiomic analysis of soft tissues sarcomas can distinguish intermediate from high-grade lesions. *Journal of Magnetic Resonance Imaging*.

[10] Zhang Y., Zhu Y., Shi X. (2019). Soft tissue sarcomas: preoperative predictive histopathological grading based on radiomics of MRI. *Academic Radiology*.

[11] Fedorov A., Beichel R., Kalpathy-Cramer J. (2012). 3D slicer as an image computing platform for the quantitative imaging network. *Magnetic Resonance Imaging*.

[12] Van Griethuysen J. J. M., Fedorov A., Parmar C. (2017). Computational radiomics system to decode the radiographic phenotype. *Cancer Research*.

[13] Shi B., Grimm L. J., Mazurowski M. A. (2018). Prediction of occult invasive disease in ductal carcinoma in situ using deep learning features. *Journal of the American College of Radiology*.

[14] Pedregosa F., Varoquaux G., Gramfort A. (2011). Scikit-learn: machine learning in Python. *Journal of Machine Learning Research*.

[15] Galant J., Martí-Bonmatí L., Sáez F., Soler R., Alcalá-Santaella R., Navarro M. (2003). The value of fat-suppressed T2 or STIR sequences in distinguishing lipoma from well-differentiated liposarcoma. *European Radiology*.

[16] Ohguri T., Aoki T., Hisaoka M. (2003). Differential diagnosis of benign peripheral lipoma from well-differentiated liposarcoma on MR imaging:is comparison of margins and internal characteristics useful?. *American Journal of Roentgenology*.

[17] Burt A. M., Huang B. K. (2017). Imaging review of lipomatous musculoskeletal lesions. *SICOT-J*.

[18] Skrzynski M. C., Biermann J. S., Montag A., Simon M. A. (1996). Diagnostic accuracy and charge-savings of outpatient core needle biopsy compared with open biopsy of musculoskeletal tumors. *The Journal of Bone & Joint Surgery*.

[19] Thornhill R. E., Golfam M., Sheikh A. (2014). Differentiation of lipoma from liposarcoma on MRI using texture and shape analysis. *Academic Radiology*.

[20] Mayerhoefer M. E., Breitenseher M., Amann G., Dominkus M. (2008). Are signal intensity and homogeneity useful parameters for distinguishing between benign and malignant soft tissue masses on MR images?. *Magnetic Resonance Imaging*.

[21] Mayerhoefer M. E., Breitenseher M. J., Kramer J., Aigner N., Hofmann S., Materka A. (2005). Texture analysis for tissue discrimination on T1-weighted MR images of the knee joint in a multicenter study: transferability of texture features and comparison of feature selection methods and classifiers. *Journal of Magnetic Resonance Imaging*.

[22] Juntu J., Sijbers J., De Backer S., Rajan J., Van Dyck D. (2010). Machine learning study of several classifiers trained with texture analysis features to differentiate benign from malignant soft-tissue tumors in T1-MRI images. *Journal of Magnetic Resonance Imaging*.

[23] Xu Q. S., Liang Y. Z., Du Y. P. (2004). Monte Carlo cross-validation for selecting a model and estimating the prediction error in multivariate calibration. *Journal of Chemometrics*.

