# Supplementary material for: Radiomics and Machine Learning Differentiate Soft-Tissue Lipoma and Liposarcoma Better than Musculoskeletal Radiologists
Source: Sarcoma. 2020 Jan 7;2020:7163453. doi: 10.1155/2020/7163453 (PMC6969992; doi:10.1155/2020/7163453)
Supplement: Supplementary Materials — Table S1: extracted radiomics features. [file 7163453.f1.pdf]

First order features	GLCM features	Shape features	GLRLM features	GLSZM features	NGTDM features	GLDM features
**10 Percentile**	Autocorrelation	Elongation	Long Run Emphasis	Gray Level Variance	Contrast	Gray level nonuniformity
**90 Percentile**	Cluster Prominence	Flatness	Long Run High Gray Level Emphasis	Large Area Emphasis		
**Energy**	Cluster Shade	Maximum 2D Diameter Column	Long Run Low Gray Level Emphasis	Small Area Emphasis		
**Entropy**	Cluster Tendency	Maximum 2D Diameter Row	Low Gray Level Run Emphasis	Zone Entropy		
**Interquartile Range**	Contrast	Maximum 2D Diameter Slice	Run Entropy	Zone Percentage		
**Kurtosis**	Correlation	Maximum 3D Diameter	Run Length Non Uniformity	Zone Variance		

**Maximum**	Difference Average	Sphericity	Run Length Non Uniformity Normalized	Large Area High Gray Level Emphasis
**Mean Absolute Deviation**	Difference Entropy	Surface Area	Run Percentage	Large Area Low Gray Level Emphasis
**Mean**	Difference Variance	Surface Volume Ratio	Run Variance	Low Gray Level Zone Emphasis
**Median**	Energy	Volume	Short Run Emphasis	Size Zone Non Uniformity
**Minimum**	Entropy	Least Axis	Short Run High Gray Level Emphasis	Small Area High Gray Level Emphasis
**Range**	Inverse Difference	Major Axis	Short Run Low Gray Level Emphasis	Small Area Low Gray Level Emphasis

**Robust Mean Absolute Deviation**	Inverse Difference Moment	Minor Axis	Gray Level Non Uniformity
**Root Mean Squared**	Inverse Difference Moment Normalized		Gray Level Variance
**Skewness**	Inverse Difference Normalized		High Gray Level Run Emphasis
**Total Energy**	Informal Measure of Correlation		High Gray Level Zone Emphasis
**Uniformity**	Informal Measure of Correlation		
**Variance**	Inverse Variance		
	Maximum Probability		

Sum Average

Sum Entropy
Sum Squares
Joint Average
Joint Energy
Joint Entropy

GLCM = gray level co-occurrence matrix

GLSZM = gray level size zone matrix

GLRLM = gray level run length matrix

NGTDM = neighboring gray tone difference matrix

GLDM = gray level dependence matrix

Table S1: Extracted Radiomics Features.
